# Development and Digital Light Processing 3D Printing of a Vitrimer Composed of Glycerol 1,3-Diglycerolate Diacrylate and Tetrahydrofurfuryl Methacrylate

**DOI:** 10.1021/acsapm.3c01018

**Published:** 2023-08-21

**Authors:** Sigita Grauzeliene, Anne-Sophie Schuller, Christelle Delaite, Jolita Ostrauskaite

**Affiliations:** † Department of Polymer Chemistry and Technology, Kaunas University of Technology, Radvilenu Road 19, LT-50254 Kaunas, Lithuania; ‡ Laboratoire de Photochimie et d’Ingénierie Macromoléculaires—EA4567, Université de Haute Alsace, Université de Strasbourg, 3b Rue Alfred Werner, 68093 Mulhouse Cedex, France

**Keywords:** vitrimer, glycerol, biobased, shape memory, weldability, repairability, DLP 3D printing

## Abstract

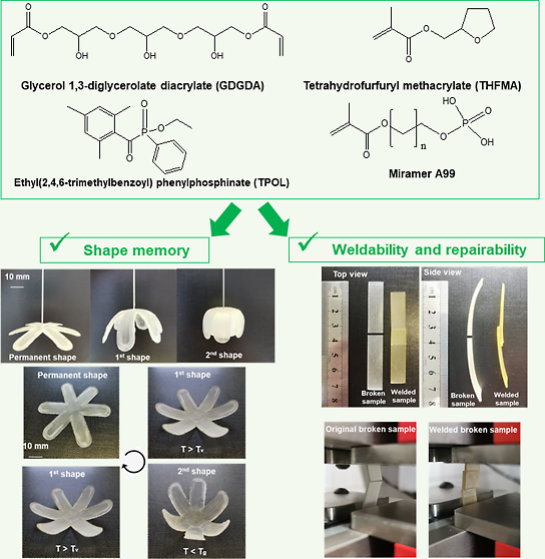

The development of biobased reshapable and repairable vitrimers has received extensive attention due to the growing focus on an environmentally friendly society. Therefore, the objective of this research was to synthesize sustainable polymers with an environmentally friendly strategy combining the benefits of renewable resources, UV curing, and vitrimers. Two biobased monomers, glycerol 1,3-diglycerolate diacrylate and tetrahydrofurfuryl methacrylate, were chosen for the preparation of UV-curable resins and tested by real-time photorheometry and RT-FTIR spectroscopy to determine their suitability for digital light processing (DLP) 3D printing. DLP 3D-printed polymer showed shape memory, weldability, and repairability capabilities by triggering the dynamic transesterification process at high temperatures. The vitrimer with a weight ratio of 60:40 of glycerol 1,3-diglycerolate diacrylate and tetrahydrofurfuryl methacrylate showed shape memory properties with a recovery ratio of 100% and a 7-fold improved tensile strength compared to the original sample, confirming efficient weldability and repairability.

## 1 Introduction

Vitrimers are a type of plastic that, unlike traditional thermoplastics and thermosets, can be reshaped and repaired without losing their original properties^1^ and has the potential to reduce plastic waste. Vitrimers are a promising class for the development of sustainable materials for a wide range of applications, including functionally tunable devices, the automotive industry, soft robotics, and aerospace.^2−6^ Vitrimers have several advantages over traditional polymers:^7−10^

- Recyclability: Vitrimers can be recycled because of the reversible dynamic covalent cross-links that respond to specific stimuli, such as heat, light, or pH. Furthermore, they can be reshaped, thus contributing to reduced waste compared to traditional polymers that can only be downcycled.
- Repairability: Vitrimers can be repaired after cracking and can have different structures, extending their durability and lifespan.
- Chemical resistance: Compared to traditional polymers, vitrimers have superior chemical resistance, making them suitable for use in harsh chemical environments.
- Improved mechanical properties: Vitrimers have been shown to have improved mechanical properties compared to traditional polymers, including high toughness and resilience, making them suitable for use in demanding applications.
- Cost-effective: When considering their improved durability, versatility, and recyclability, vitrimers are frequently cost-competitive with traditional polymers.
- Eco-friendly: Vitrimers can be produced from renewable resources, and their ability to be recycled and reshaped for multiple cycles reduces waste and the need for raw materials compared to traditional polymers.

Dynamic covalent bonds can be created by numerous reversible reactions, including disulfide, transamination, transcarbonylation, imine exchange, urethane reversion, vinylogous urethane exchange, etc.,^11,12^ but the thermoactivated transesterification reaction of the hydroxyl and ester groups is the most used reaction in optical 3D printing.^13^ However, many of the monomers used were petroleum-based, for example, the most widely known bisphenol A epoxy resin,^14^ and the use of biobased monomers for vitrimer synthesis represents one of the most important contributions of modern polymer science solving the problem of the use of petroleum-based materials. Shape memory polymers (SMPs) as a class of stimulus-responsive polymers can return to their permanent shape from a programmed temporary shape under external stimuli, such as light, heat, magnetism, and electricity, and have a broad application in soft robotics, flexible electronics, biomedical devices, and aerospace engineering.^15^ The mostly reported SMP class includes thermo-responsive SMPs. The switching characteristic for these polymers is usually a direct change in temperature that is based on either glass transition temperature (*T*_g_), for amorphous polymers, or *T*_g_ and topology freezing temperature (*T*_v_), for vitrimers, named shape memory vitrimers (SMVs).^16^

**Figure 1:**
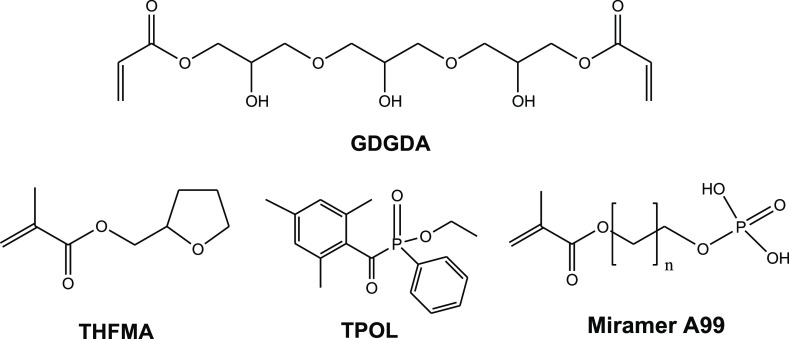
Chemical structures of GDGDA, THFMA, TPOL, and Miramar A99.

Herein, the development and investigation of a new biobased transesterification vitrimer suitable for digital light processing (DLP) 3D printing are reported. Vegetable oils, lignin, carbohydrates, natural carboxylic acids, natural rubbers, and latex have been used to synthesize biobased transesterification vitrimers, but most of them were epoxy-based.^17−20^ However, the most commonly used resins for DLP are acrylate- and methacrylate-based due to their high polymerization rate and commercially available monomers.^21^ Therefore, glycerol 1,3-diglycerolate diacrylate (GDGDA) (Figure 1) was selected as a monomer as it has a fragment of glycerol in the backbone, which is a byproduct of the biodiesel production from vegetable oils and animal fats,^22−24^ apart from having the hydroxyl and ester groups required for the transesterification reaction. In addition, glycerol has very low toxicity and is biodegradable,^25^ and glycerol compounds such as GDGDA and 2-hydroxy-3-phenoxypropyl acrylate^26^ have been used for vitrimer synthesis. GDGDA is commercially available and has been used for vitrimer DLP 3D printing.^27−31^ Biobased monomer tetrahydrofurfuryl acrylate (THFA), which is derived from agricultural waste, including corn cobs and sugarcane bagasse, was used in vitrimer synthesis together with synthetic monomers, such as acrylamide.^32^ However, acrylamide is one of the substances of great concern, which implies that the industry must follow strict regulations to control the residual monomers in the product.^33^ The use of tetrahydrofurfuryl methacrylate (THFMA) in the development of biobased vitrimers for DLP 3D printing is reported here for the first time. THFMA is superior compared to THFA in that it is less reactive and its incorporation into resins can enhance tensile strength and rigidity.^34^ In order to work at a curing wavelength of 385 nm with a DLP 3D printer, ethyl (2,4,6-trimethylbenzoyl)phenylphosphinate (TPO-L) was chosen as the photoinitiator because it is a liquid, easily mixed with monomers, and has a maximum absorption wavelength at 383 nm.^35^ The chosen transesterification catalyst Miramar A99 has properties such as good solubility in acrylate monomers, promotion of fast stress relaxation of materials at high temperatures, and covalent incorporation into the photopolymer network.^28,29^ In this study, the biobased monomers GDGDA and THFMA were mixed in various solvent-free ratios with a photoinitiator ethyl(2,4,6-trimethylbenzoyl)phenylphosphinate (TPOL) and a Miramar A99 transesterification catalyst. For the first time, the photocuring kinetics, rheological properties, and conversion of double bonds of acrylate of resins based on GDGDA and THFMA were investigated. It was determined that the concentration of THFMA affected the rheological and viscosity parameters. Stress relaxation studies revealed that THFMA is an appropriate monomer for GDGDA UV curing because, after this process, the dynamic networks can quickly undergo thermoactivated network topology changes. A new resin based on GDGDA and THFMA was used in DLP 3D printing to produce vitrimeric 3D structures. The weldability, repairability, and shape memory features of the vitrimer were achieved by the addition of THFMA.

## 2 Materials and Methods

### 2.1 Materials

GDGDA was purchased from Merck, Darmstadt, Germany. THFMA and TPOL were purchased from Sartomer (Arkema Group, Colombes Cedex, France). Miramar A99 was purchased from Miwon Europe GmbH, Mainz, Germany. All chemicals were used as received.

### 2.2 UV-Curable Resin Preparation

GDGDA and THFMA were mixed in various ratios at room temperature to prepare the resins (Table 1). 3 mol % of TPOL as the photoinitiator and 5 wt % Miramer A99 as the transesterification catalyst were added to the resins. Resin codes are composed of a number that expresses the amount and the first letter of the monomer abbreviation, e.g., 60G/40T is a resin with a weight ratio of GDGDA and THFMA of 60:40, respectively.

**Table 1:** Composition and Viscosity of Resins

resin	amount of GDGDA (wt %)	amount of THFMA (wt %)	amount of TPOL (mol %)	amount of Miramar A99 (wt %)	viscosity (mPa·s)
100G	100		3	5	68,618 ± 3417
80G/20T	80	20			9354 ± 240
60G/40T	60	40			1394 ± 69
40G/60T	40	60			274 ± 4
20G/80T	20	80			67 ± 3
100T		100			32 ± 2

An Anton Paar MCR302 rheometer (Graz, Austria) with a plate/plate accessory (15 mm top plate diameter), a shear rate range of 0.001– 50 s^–1^, and 25 °C was used to measure the viscosity of the resins.

### 2.3 DLP 3D Printing

An Asiga MAX UV 385 3D printer (Sydney, Australia) was used to print rectangular samples (70 × 10 × 1) ± 0 mm and the structure of the “gripper” (diameter 50 mm, thickness 1 mm). The first 200 μm layer was exposed for 9 s, while the other 100 μm layers were exposed for 3.4 s to irradiation with an intensity of 9.8 mW/cm^2^ at a wavelength of 385 nm.

### 2.4 Characterization Techniques

Real-time Fourier transform infrared spectroscopy (RT-FTIR) was used to monitor the double bond conversion (DBC) of the acrylate. Measurements were performed with a Bruker Vertex 70 spectrometer with a spectral resolution of 4 cm^–1^ and 30 scans. The sample was analyzed in laminar mode between two internal 10 μm polypropylene pellets separated by a 25 μm Teflon spacer. The samples were exposed under a 385 nm LED (UWave UFIBER) with an irradiance of 9.8 mW/cm^2^.

An Anton Paar MCR302 rheometer (Graz, Austria) with a plate/plate accessory and OmniCure S2000 UV curing equipment (Lumen Dynamics Group Inc. Mississauga, Ontario, Canada) were used to investigate the photocuring kinetics of the resins. The diameters of the bottom glass plate and the top steel plate were 38 and 15 mm, respectively. The tests were carried out at a temperature of 25 °C and a resin layer thickness of 100 μm. The resin was irradiated with UV light with a wavelength of 320–390 nm and an intensity of 9.3 W/cm^2^ through the lower bottom glass plate. A shear test was performed at a frequency of 5 Hz and a strain of 1%. The storage modulus *G*′, loss modulus *G*″, and the complex viscosity η* were determined after 120 s of the resin irradiation. The intersection point of *G*′ and *G*″ was determined as the gel point *t*_gel_. The shrinkage was calculated as the gap difference before and after UV curing.

Polymer samples (0.2 g) were extracted with acetone in a Soxhlet extractor for 24 h to determine the insoluble fraction. The yield of the insoluble fraction was calculated as the difference between the weights of the polymer sample before and after extraction and drying.

An Anton Paar MCR302 rheometer (Graz, Austria) was used to determine the topology freezing temperature (*T*_v_) using stress relaxation experiments. Stress reduction over time was recorded by applying a 5% step strain and a temperature of 180–220 °C.

The glass transition temperature (*T*_g_) was determined by dynamic mechanical thermal analysis (DMTA) with an Anton Paar MCR302 rheometer (Graz, Austria). A sample with dimensions of (10 × 10 × 1) ± 0.01 mm was tested in shear mode from −15 to 70 °C at a rate of 2 °C/min. *T*_g_ was defined as the tan δ curve peak.

A PerkinElmer TGA 4000 equipment (Llantrisant, UK) was used to determine the thermal stability of the polymers. A heating rate of 20 °C/min and a nitrogen flow rate of 100 mL/min were used.

### 2.5 Shape Memory Experiments

The 3D-printed sample of the “gripper” (50 mm in diameter, 1 mm thick) was examined for shape memory properties. The first shape was obtained by heating the sample above *T*_v_ at 80 °C and transforming it into the desired shape. The second shape was obtained by cooling the sample down to 40 °C (above *T*_g_), transforming, and cooling it below room temperature in an ice bath (below *T*_g_). The sample reached its permanent shape when heated again above its *T*_g_.

The shape fixity (SF) and recovery ratios (RR) were calculated as the difference between the polymer sample lengths at various stages of the shape memory test (according to eqs 1 and 2, respectively).^36^ The sample with its original length *L*_0_ (cm) was first heated above *T*_v_ to 80 °C and elongated to a length *L*_s_ (cm), which was 8 cm. The tensile load was applied to the sample while it was in the fixing stage, the sample was cooled to ambient temperature, and then the load was released and the length was reduced from *L*_s_ to *L*_f_. The deformed sample was then heated to 80 °C to allow the length *L* (cm) to recover.
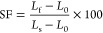
1
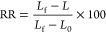
2

### 2.6 Weldability and Repairability Experiments

A Testometric M500-50CT machine (Testometric Co Ltd, Rochdale, U.K.) with HDFF100 grips was used to test the mechanical properties of rectangular welded samples with the size of (70 × 10 × 1) ± 0 mm, which were cut into two equal pieces, brought into contact with each other (no pressure was applied), and heated at 180 °C over 5 h to obtain the repaired sample. Elongation at break, tensile strength, and elastic modulus were determined according to ISO 527-3. Samples were analyzed by applying a cross-head speed of 5 mm/min. An average of three parallel measurements were calculated. The experimental results of the group varied by no more than 5%.

## 3 Results and Discussion

### 3.1 Selection of the Resin for Vitrimer Synthesis

**Figure 2:**
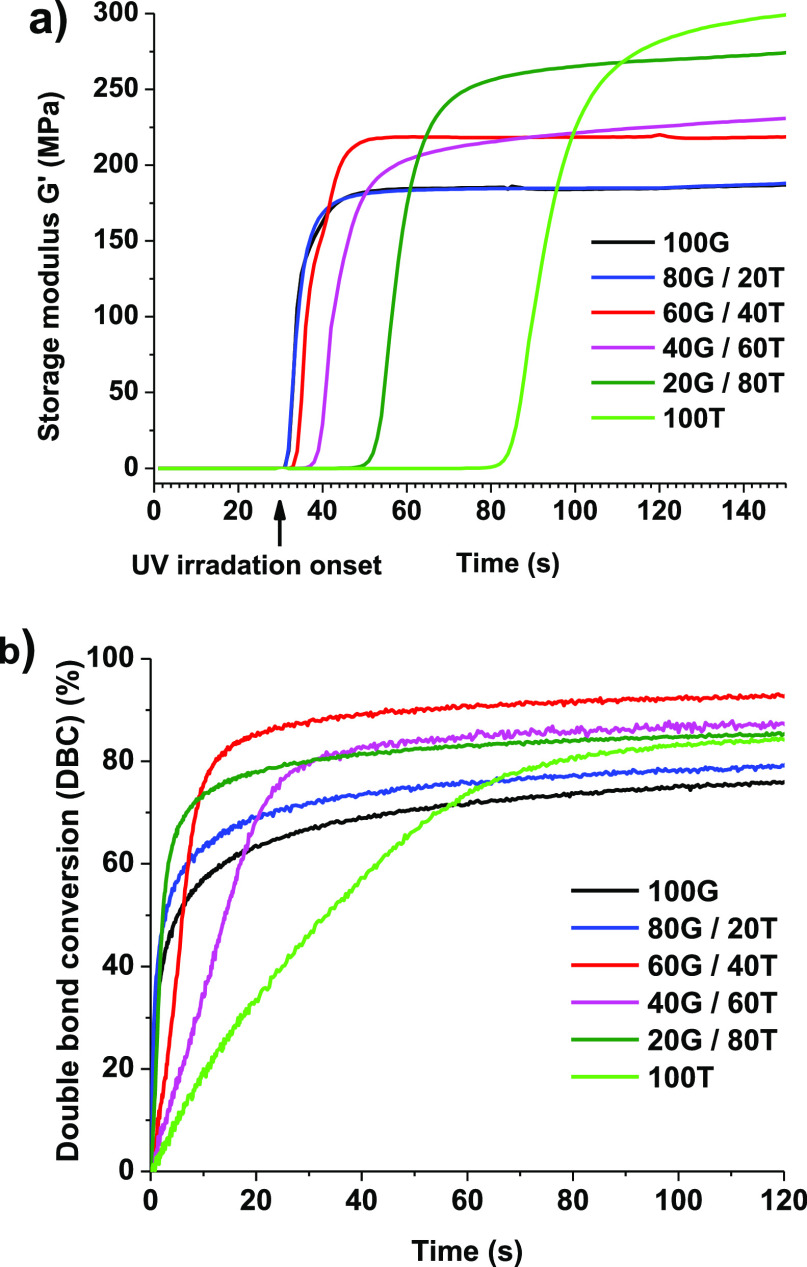
Storage modulus *G*′ (a) and DBC (b) curves versus irradiation time of resins.

Real-time photorheometry and RT-FTIR techniques were used to determine the optimal resin for vitrimer DLP 3D printing. Photorheometry is a crucial instrument for monitoring rheological parameters such as viscosity, shrinkage, and rigidity of the material.^37^ This analysis is very informative when the resin is solidified during the 3D printing process. The viscosity of conventional resins for DLP 3D printing typically ranges from 200 to 1500 mPa·s.^38^ The viscosity of the resins based on GDGDA and THFMA ranged from 32 to 68,618 mPa·s (Table 1) and reduced with the addition of THFMA. These resins were kept in the dark for 3 months, but there were no notable changes in viscosity. Other rheological characteristics such as the storage modulus (*G*′), the loss modulus (*G*″), the complex viscosity (η*), the shrinkage, and the gel point are listed in Table 2. The addition of THFMA to the resins increased the rigidity of the polymers as the values of *G*′ (Figure 2a), *G*″, and η* increased with the increasing amount of THFMA as a result of the densely cross-linked network. However, the shrinkage and the gel point values also increased with the addition of THFMA. Under irradiation, a fast conversion of acrylic double bonds (DBC) was determined and led to 91.7% of conversion (Figure 2b). The DBC was increased up to 40 wt % concentration of THFMA and then decreased gradually. The reason was that at higher concentrations of THFMA, radicals and monomers are trapped in the vitrified medium and the final DBC is reduced.^39^ The resin 60G/40T was selected for DLP 3D printing as it exhibited the highest DBC (91.7%), suitable viscosity (1394 mPa·s), low gel point time (2 s), and the abundance of hydroxyl and ester groups, which contribute to transesterification reactions. Furthermore, the topological freezing transition temperature (*T*_v_) and other thermal characteristics of the polymer 60G/40T were determined, as well as shape memory and weldability properties.

**Table 2:** Data of the Real-Time Photorheometry and DBC of the Resins

resin	storage modulus *G*′ (MPa)	loss modulus *G*″ (MPa)	complex viscosity η* (GPa·s)	shrinkage (%)	gel point *t*_gel_ (s)	DBC (%)
100G	181.2 ± 5.8	122.7 ± 2.6	7.4 ± 0.5	9.0 ± 1.0	1.0 ± 0.0	76.0 ± 3.4
80G/20T	190.0 ± 1.5	130.3 ± 3.8	7.8 ± 0.3	13.5 ± 0.5	1.0 ± 0.0	79.5 ± 2.0
60G/40T	224.5 ± 5.8	134.5 ± 1.6	8.4 ± 0.5	15.5 ± 0.5	2.0 ± 0.0	91.7 ± 2.2
40G/60T	230.9 ± 12.0	138.7 ± 1.4	8.7 ± 0.4	16.0 ± 1.0	4.0 ± 0.0	87.1 ± 0.6
20G/80T	269.6 ± 4.6	148.3 ± 8.8	10.1 ± 0.5	16.5 ± 0.5	13.5 ± 0.5	85.5 ± 0.8
100T	299.1 ± 14.6	164.7 ± 2.1	15.1 ± 0.4	18.5 ± 1.5	59.5 ± 0.5	81.8 ± 3.6

### 3.2 Characterization of the Cross-Linked Polymer Structure

**Figure 3:**
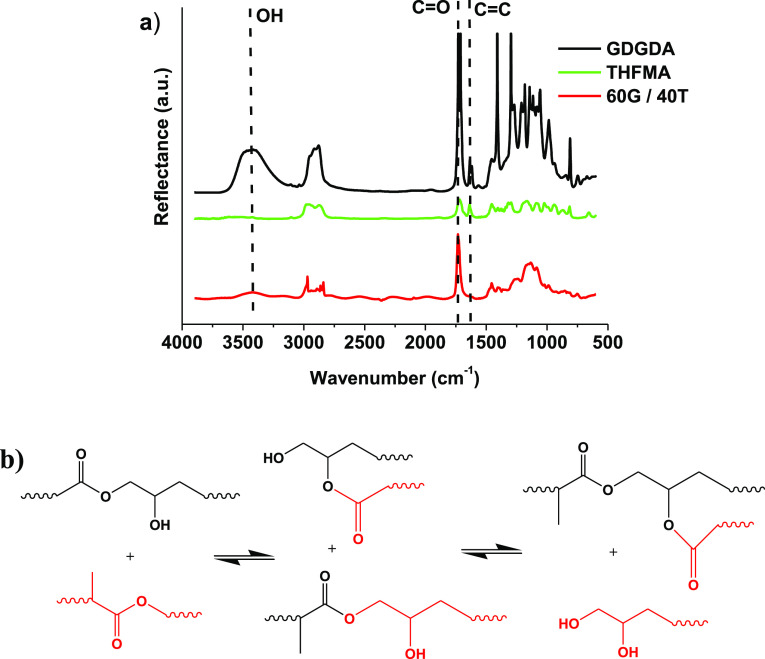
FTIR spectra of GDGDA, THFMA, and the DLP 3D-printed sample of polymer 60G/40T (a) and the possible transesterification mechanism (b).

The chemical structure of the 60G/40T polymer was confirmed by RT-FTIR spectroscopy (Figure 3). The intensity of the C=C group signal, which was present at 1638 cm^–1^ in the RT-FTIR spectra of GDGDA and THFMA, decreased in the polymer spectrum. This suggested that a polymer network has been formed. The intensities of the OH and C=O group signals at 3474 and 1745 cm^–1^ were reduced in the polymer spectrum compared to the spectra of the starting materials, but remained, which is essential for the transesterification reaction. Transesterification is a well-known chemical reaction in which an ester group is exchanged with the hydroxyl group. A possible transesterification mechanism of GDGDA and THFMA is shown in Figure 3b. The ester groups of THFMA are exchanged by hydroxyl groups of GDGDA.

### 3.3 Stress Relaxation

**Figure 4:**
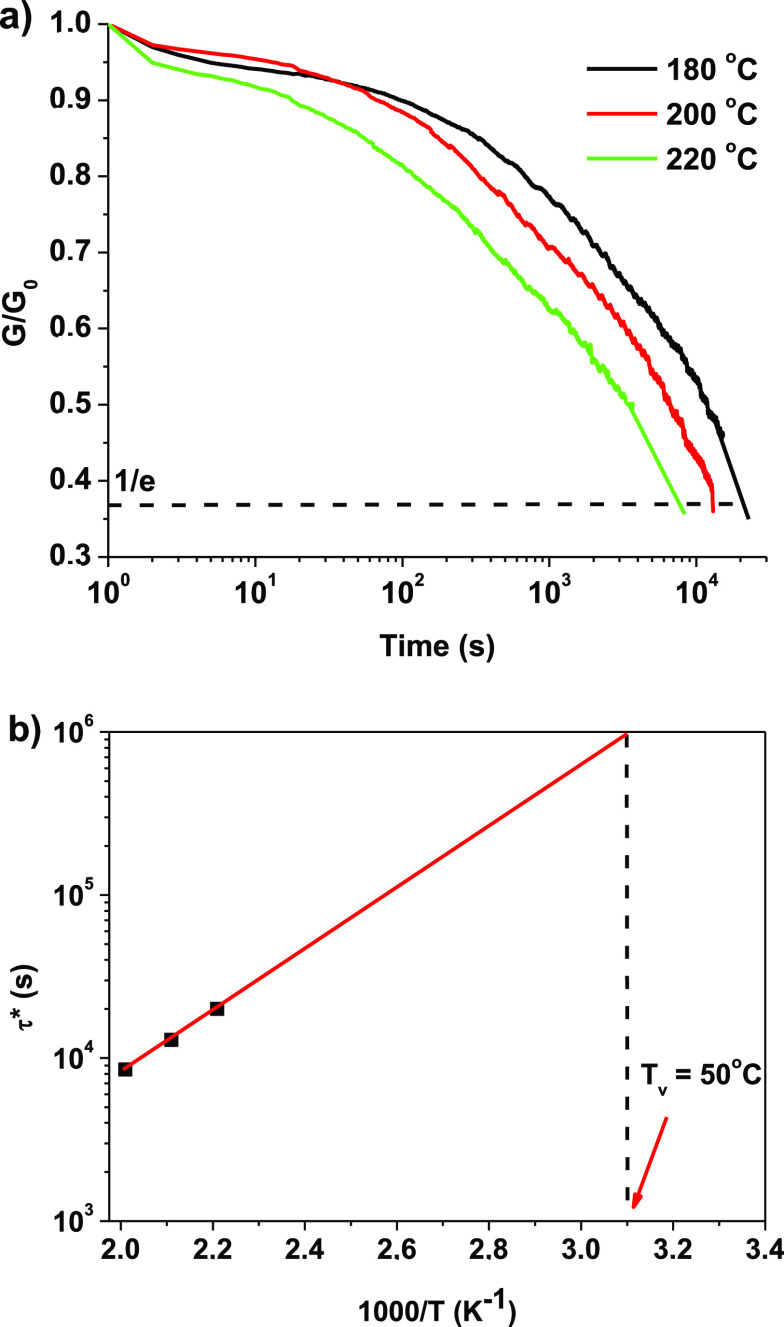
60G/40T stress relaxation curves versus time (a) and the Arrhenius plot of relaxation times (b).

**Figure 5:**
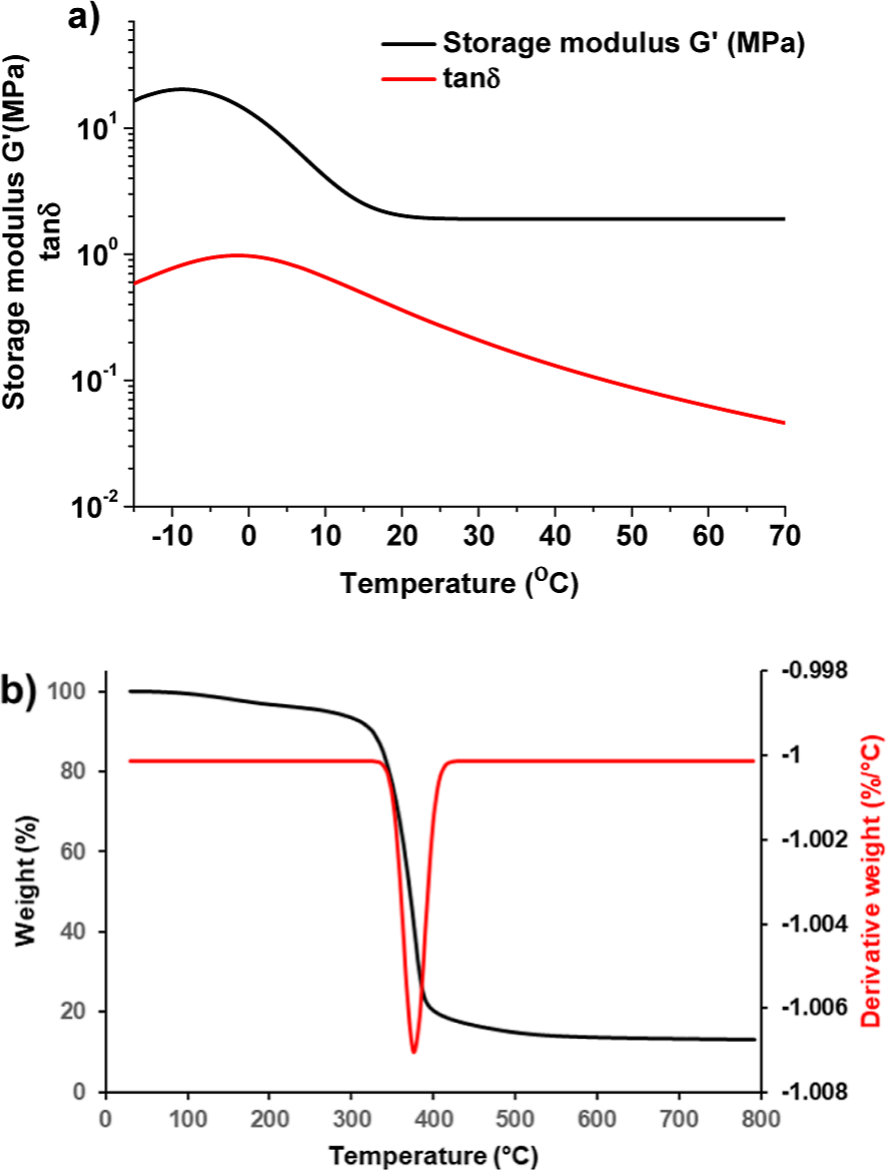
Curves of the storage modulus *G*′ and tan δ versus temperature (a), and thermogravimetric curves of the polymer 60G/40T (b).

The design of vitrimers is based on the reversible network topology freezing of atoms that are covalently bound to create a network.^40^ The material undergoes stress relaxation and flows when the network can change its topology through bond exchange reactions, even if the total number of bonds remains constant over time. The exchange reactions in the vitrimer are thermally triggered. Due to this, stress relaxation tests of polymer 60G/40T were carried out at temperatures ranging from 180 to 220 °C, and *T*_v_ was identified, at which the transition from viscoelastic solid to viscoelastic liquid occurs (Figure 4). Thermal degradation was avoided because the 60G/40T sample had thermal stability greater than 220 °C (Figure 5b). The time at which the sample relaxes to 1/*e* of the original modulus is known as the relaxation time (τ*) and can be gathered from stress relaxation curves.^41^ The 60G/40T polymer can relieve stress in the temperature range of 180–220 °C, as shown in Figure 4a, and the time required to do that was reduced from 5.5 to 2.3 h, as a result of dynamic bond exchange and chain diffusion. Vitrimers exhibit thermoset behavior below *T*_v_, whereas above *T*_v_, exchange reactions are fast and allow flow-through reversible reactions. By extrapolating the data to a relaxation time of 10^6^, *T*_v_ can be calculated.^40^ Consequently, the *T*_v_ of the 60G/40T vitrimer was calculated to be 50 °C from the Arrhenius curve.

### 3.4 Thermal Properties

The thermal properties of the polymer 60G/40T were investigated by DMTA and TGA. *T*_g_, defined as the tan δ curve peak (Figure 5a), was 0 °C due to long and flexible alkyl chains^42^ of GDGDA fragments in the polymer. However, this polymer is a solid material at room temperature. The relaxation of the polymer takes place sharply with a notable change in the storage moduli decrease from the glass to the rubbery state, and the storage share modulus in the rubbery plateau region (*G*_r_′) was 0.05 MPa. The thermal decomposition of the polymer 60G/40T proceeded in one step (Figure 5b), which confirmed a densely cross-linked network, and the decomposition temperature at a weight loss of 10% (*T*_dec-10%_) and char yield were 326 °C and 13.2%, respectively. The yield of the insoluble fraction (87.9%) confirmed the high thermal stability of the polymer. The derivative weight curve shows that degradation begins at 350 °C and reaches a maximum rate above 390 °C.

### 3.5 Shape Memory Properties

**Figure 6:**
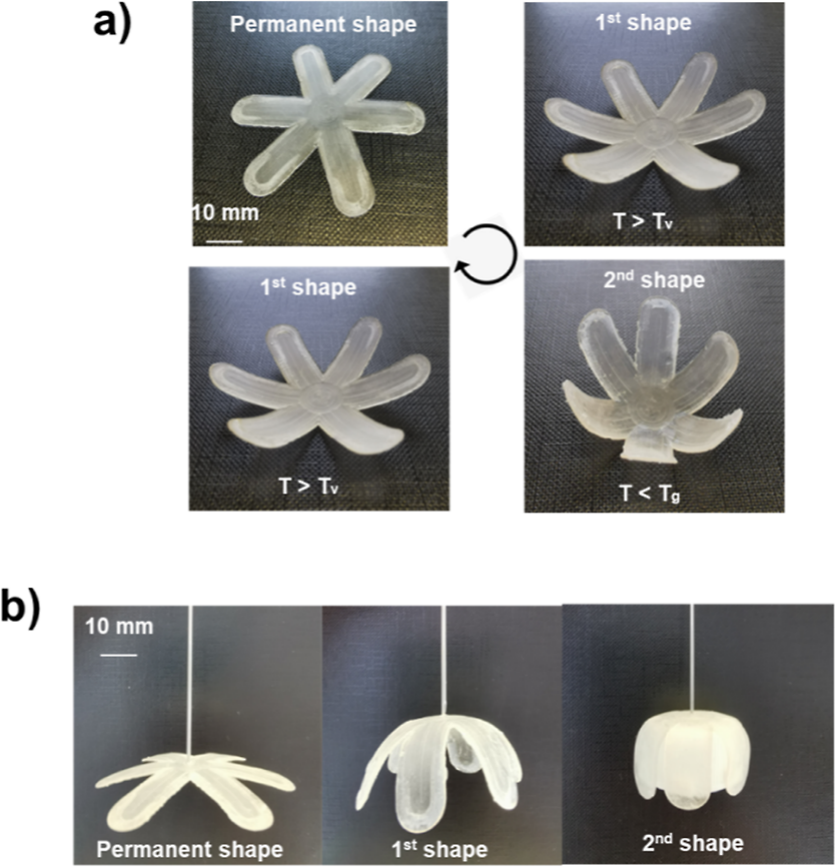
Photographs (a,b) expressing the monitoring of the shape memory behavior of the DLP printed “gripper” sample.

Figure 6 shows the two-way shape memory cycle of the DLP 3D printed “gripper” sample 60G/40T. The first temporary shape was fixed by heating the sample above the *T*_v_ (50 °C) and then transforming it into the desired shape with an external force. When the temperature exceeds *T*_v_, polymer chains exchange quickly and polymer networks behave like fluids, and the plasticity of the vitrimer networks becomes noticeable.^1,3^ Therefore, shape programming of SMVs at a temperature above *T*_v_ that uses a reversible glass transition as the temporary fixity may reduce the shape recovery. As a result, the “gripper” sample was cooled to 40 °C, which is higher than *T*_g_. The second temporary shape of the sample was fixed by cooling it below room temperature. When the sample was heated, the two shapes were consistently recovered. The “gripper” sample had a 100% RR, which means that the sample can recover its original length, and a 100% SF that shows no shrinkage after the load is removed. Due to its excellent shape memory property, the 60G/40T vitrimer is a candidate for multifunctional devices in robotics. These robots have the ability to perform a wide range of complex tasks, including drug delivery, search and rescue operations, and acting as human assistants.^43^

### 3.6 Weldability and Repairability Properties

**Figure 7:**
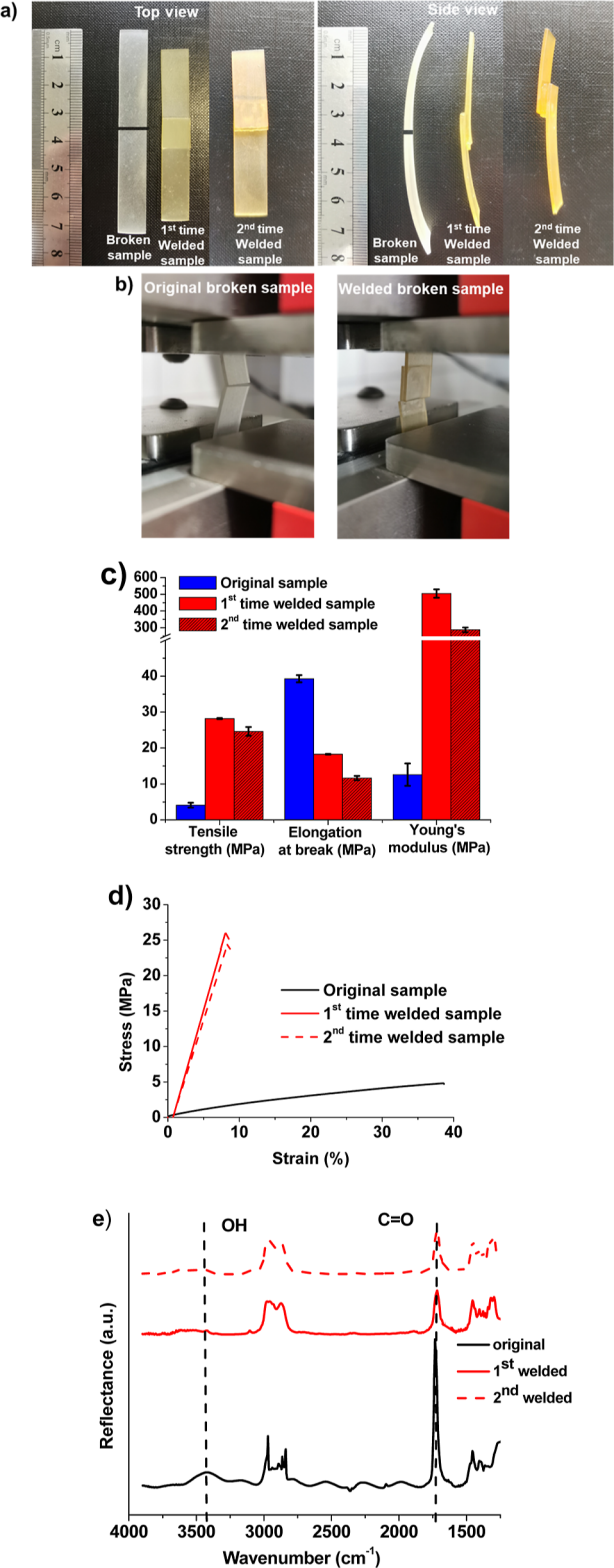
Pictures (a,b), mechanical characteristics values (c), stress–strain curves (d), and FTIR spectra (e) of the original and repaired 60G/40T samples.

The interest in repairable materials that increase the lifetime of products ranges from protective coatings to the biomedical industrial area.^43^ The diffusion of polymer chains through the interface in close proximity to *T*_g_ gives thermoplastics their unique ability to be welded.^44^ However, thermosets cannot be reprocessed after an irreversible curing reaction occurs. In this case, vitrimers as intermediate materials between thermoplastics and thermosets, obtained through a dynamic transesterification reaction, could have weldability and repairability properties. Therefore, to investigate the repairability properties, the 3D-printed rectangular sample 60G/40T was cut into two equal parts, adhered together with a 10 mm overlap, and rejoined at 180 °C during 5 h (Figure 7a) for the first and second time, and the mechanical properties of the original and welded samples were compared. As shown in Figure 7b, the tensile testing of the sample revealed that the rupture occurred at a different position from that of the welded area, demonstrating that the welded area could withstand greater stretching loads than other locations. After thermal treatment, the mechanical properties were improved, as the values of tensile strength and Young’s modulus were increased (Figure 7c), which means that additional hydrogen bond formation occurred during the curing at 180 °C. The sample loses its elasticity, as can be seen from the side view photograph, and elongation at break of the welded sample was recovered by 46.6%. Young’s modulus and tensile strength were improved 40 and 23, and 7 and 6 times, respectively, compared to those of the original sample (Figure 7d) due to hydrogen bonding formation. This was confirmed by the FTIR spectra of samples before and after the first and second welding. After heating at 180 °C, a decrease in OH and C=O groups was observed due to hydrogen bond formation (Figure 7e).^45^ This experiment showed excellent repairability of the cured sample 60G/40T, indicating a reversible transesterification exchange reaction and a topological network rearrangement once the sample parts are in contact.

## 4 Conclusions

A GDGDA- and tetrahydrofuryl methacrylate-based transesterification vitrimer suitable for DLP 3D printing and having shape memory, weldability, and repairability properties was developed. For DLP 3D printing, the resin with the maximum DBC (91.7%), suitable viscosity (1394 mPa·s), low gel point (2 s), and a high concentration of hydroxyl and ester groups supporting transesterification processes was chosen. THFMA is a suitable monomer for the UV curing reactions of GDGDA, according to stress relaxation experiments since dynamic networks can rapidly undergo thermoactivated network topology changes following UV curing, and the topological freezing transition temperature was calculated to be 50 °C. In UV curing of GDGDA, THFMA was found to be an appropriate cross-linker as it showed shape memory, weldability, and repairability qualities. The synthesized vitrimer could be used in robotics to perform a variety of sophisticated activities such as drug distribution, search and rescue missions, and serving as human assistants.
